# Application of high-quality targeted temperature management guided by multimodal brain monitoring in brain protection of patients with cardiac arrest: A case series

**DOI:** 10.1097/MD.0000000000040943

**Published:** 2024-12-20

**Authors:** Weiping Xia, Meiling Ai, Xinhua Ma, Chenhuan Hu, Qianyi Peng, Chunguang Zhao, Qi Liu, Shixiong He, Li Huang, Lina Zhang

**Affiliations:** a Department of Intensive Care Medicine, Xiangya Hospital, Central South University, Changsha, China; b National Clinical Research Center for Geriatric Disorders, Xiangya Hospital, Central South University, Changsha, China.

**Keywords:** brain function monitoring, cardiac arrest, prognosis, targeted temperature management

## Abstract

**Rationale:**

Cardiac arrest (CA) is an acute emergency with high mortality and is closely associated with the risk of brain damage or systemic ischemia–reperfusion injury, post-traumatic stress symptoms.

**Patient concerns:**

Targeted temperature management in the intensive care unit can improve the neurological outcomes of patients who are comatose after resuscitation from CA. However, there is often a lack of specific evaluation methods for optimal target temperature settings.

**Diagnoses:**

From November 2021 to October 2022, 9 CA patients received prompt cardiopulmonary resuscitation and return of spontaneous circulation after approximately 10 to 30 minutes of cardiopulmonary resuscitation in Xiangya Hospital, Central South University.

**Interventions:**

We retrospectively reviewed 9 CA patients’ medical data, including demographic characteristics, hemodynamic change, clinically relevant score, imageological examination, transcranial Doppler ultrasonography, electroencephalogram (EEG), somatosensory-evoked potential, and laboratory data.

**Outcomes:**

According to the result of each patient’s transcranial Doppler ultrasonography, somatosensory-evoked potential, and EEG to formulate an individualized target temperature. Contrary to the internationally recommended target of hypothermia, we found that not all patients require hypothermia therapy to maintain normal cerebrovascular autonomic regulation function. And neuron-specific enolase and S100β in patients showed a downward trend after hypothermia therapy. Compared with before hypothermia treatment, clinically relevant scores were reduced in patients with good prognosis. Intracranial congestion or ischemia was improved and intracranial pressure was reduced in all patients during hypothermia treatment. For patients with good EEG response, the ratio of gray matter in the brain increased and the neurological prognosis was significantly improved. Finally, after 6 months of follow-up, we found 3 patients died and 1 patient had a long-term vegetative state, the other patients had a good prognosis.

**Lessons:**

Individualized targeted temperature management under the guidance of multimodal brain monitoring plays an important role in brain protection of patients with CA.

## 1. Introduction

Cardiac arrest (CA) is an acute emergency with high mortality and is closely associated with the risk of neurological injury. Despite the widespread use of cardiopulmonary resuscitation (CPR) and automated external defibrillator devices in China, CA is still an important cause of morbidity and mortality, and one of the causes of death worldwide. In China, 550,000 individuals suffer from CA every year. The survival rate of out-of-hospital cardiac arrest (OHCA) is less than 1% and in-hospital CA is less than 9.1% in China, both lower than that of the United States.^[1]^ While most CA patients die during an acute event, a large proportion of CA deaths occur after successful resuscitation due to the development of post-CA syndrome. For example, when spontaneous circulation is restored, ischemia-reperfusion injury occurs and induces multiple organ dysfunction, like post-hypoxic-ischemic brain injury,^[2]^ infection, and metabolic derangements.^[3]^ Therefore, identifying patients with poor neurologic outcomes can not only avoids unnecessary medical resource consumption but also allows more resources to be focused on patients with recovery potential. Post-cerebral resuscitation care is a key factor in maximizing survival and neurological outcomes.

A number of studies have found that targeted temperature management (TTM) in the intensive care unit (ICU) can improve the neurological outcomes of patients who are comatose after resuscitation from CA.^[4,5]^ At present, some researchers have found that TTM in the ICU improves the neurological outcomes of comatose patients resuscitated after CA.^[6]^ The guideline recommended 12 to 24 hours at 32°C to 36°C for high-quality TTM.^[7]^ However, there is often a lack of specific evaluation methods for optimal target temperature settings. In addition, some other studies suggest that TTM may even have potentially harmful effects^[8]^, especially at lower temperatures, including arrhythmias, electrolyte and glucose disturbances, infection, and increased risk of bleeding or circulatory instability,^[9]^ with no discernible benefit on neurological outcomes.^[10,11]^ Recently, electroencephalogram (EEG), somatosensory evoked potential (SSEP), transcranial Doppler ultrasonography (TCD), radiological imaging of computed tomography or magnetic resonance imaging of the brain, and biomarkers (e.g., NSE, S100β) of brain injury have been used to predict neurological prognostication. Due to the complexity of brain structure and function, each monitoring method has its limitations. Hence, based on the results of multimodal dynamic brain monitoring, we set the optimal target temperature to treat resuscitated patients after CA and follow-up their outcomes under the premise of ensuring adequate cerebral blood supply. We here report our experience and results through nine CA patients and discuss potential benefits.

## 2. Patient data description

This is a retrospective study of 9 CA patients treated in the ICU of the Xiangya Hospital, Central South University between November 2021 and October 2022. We retrospectively reviewed the patients’ medical data, including demographic characteristics, hemodynamic change (mean arterial pressure), clinically relevant score (Glasgow coma scale [GCS], Acute physiology, Age and Chronic health evaluation [APACHE II], sequential organ failure assessment [SOFA], Richmond agitation-sedation scale [RASS] scores), imageological examination (brain gray matter ratio), TCD, EEG, SSEP, laboratory (NSE, S100β), and diagnostic data. Combined with these monitoring methods, we built a unique multimodal monitoring system to set personalized hypothermia targets and hypothermia periods according to each patient’s specific condition.

The inclusion criteria of this study were as follows: firstly, these patients were collected from the same medical unit of our institution to reduce confounding bias. Secondly, regardless of the cause of CA, all patients received prompt CPR and return of spontaneous circulation after approximately 10 to 30 minutes of CPR. Thirdly, regardless of the in/out-of-hospital CA, all patients were older than 18 years and began cooling treatment as soon as they received emergency medical services. The exclusion criteria were as follows: pregnant woman, patients with overt cognitive impairment, history of cerebrovascular disease, acute stroke, active craniocerebral hemorrhage, known history of severe coagulopathy or continuous anticoagulant medication, CA due to drugs, known poor prearrest cerebral performance category, and known terminal disease (chronic kidney failure, tumor, and so on).

## 3. Results

The patient’s medical history at arrival in the ICU of our institution is shown in Table 1. Among the 9 patients (male:female = 8:1), 4 patients were transferred to our department after resuscitation of CA in other hospitals (cases 2, 6, 7, 9), 2 patients suffered in-hospital CA at the emergency department of our hospital (cases 1, 8), 1 patient had CA in school (case 5), 1 patient collapsed in hotel (case 4), and 1 patient suddenly lost consciousness while doing outdoor activities (case 3). All patients were intubated, and 3 of the patients had a history of hypertension, 2 patients had a history of diabetes, 1 patient had a history of syncope, and 1 patient had a history of coronary heart disease.

**Table 1:** Characteristics of patients.

Variables	Numbers (N)
Gender (M/F)	8/1
Age	43.6 ± 20.2
Out-of-hospital cardiac arrest	7
Time from collapse to ROSC	17.2 ± 5.4
History of hypertension	3
History of diabetes	2
History of coronary heart disease	1
History of syncope	1
Witnessed arrest	9

F = female, M = male, N = number, ROSC = return of spontaneous circulation.

We describe a novel personalized cooling strategy that monitors brain function in real time based on the result of each patient’s TCD, SSEP, and EEG to formulate an individualized target temperature rather than a uniform temperature. Treatment with therapeutic hypothermia was initiated in all patients with ice packs, cooling blankets, and ice-cold fluids at the earliest possible stage. While in the ICU, patients received intravenous injection infusions of remifentanil, midazolam, propofol, esketamine (cases 2, 3, 4, 5, 6), promethazine, and chlorpromazine (cases 2, 8, 9). With the exception of cases 4, 5, 6, 9, all patients were treated with muscle relaxants for preventing shivering while guided cooling to target temperature according to brain monitoring (Table 2).

**Table 2:** The summary of cooling drugs for each patients.

Medicine	Patient								
	1	2	3	4	5	6	7	8	9
Physical cooling	Y	Y	Y	Y	Y	Y	Y	Y	Y
Remifentanil	Y	Y	Y	Y	Y	Y	Y	Y	Y
Midazolam	Y	Y	Y	Y	Y	Y	Y	Y	Y
Propofol	Y	Y	Y	Y	Y	Y	Y	Y	Y
Esketamine	N	Y	Y	Y	Y	Y	N	N	N
Promethazine	N	Y	N	N	N	N	N	Y	Y
Chlorpromazine	N	Y	N	N	N	N	N	Y	Y
Muscle relaxants	Y	Y	Y	N	N	N	Y	Y	N

N = no, Y = yes.

**Figure 1.**
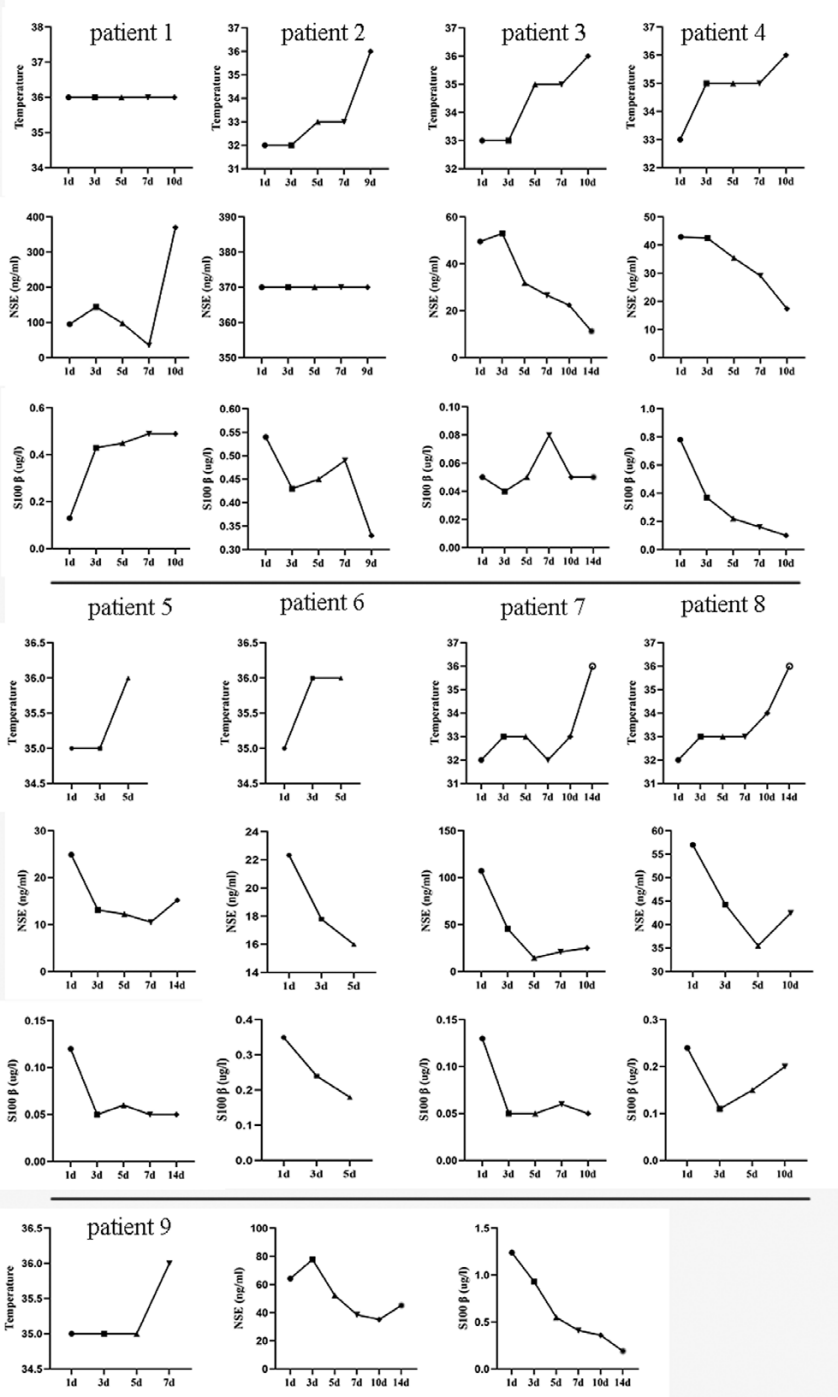
The dynamic changes of targeted temperature management, NSE, and S100 β in each patient.

As shown in Figure 1, according to the dynamic recording of TCD, SSEP, and EEG changes of each patient, the TTM management (32°C–36°C) of each patient was dynamically adjusted in real time to ensure normal brain function of the patient. Different from the internationally recommended target of 32°C to 34°C hypothermia, we found that some patients (cases 1, 5, 6, 9) maintained normal brain function even without hypothermia or slightly below normal body temperature. Furthermore, it can be seen from Figure 1 that after hypothermia treatment, the dynamic changes of NSE and S100 β in patients showed a downward trend. Table 3 records the changes in GCS, APACHE II, SOFA, RASS scores of all patients within 24 hours before hypothermia and within 24 hours after hypothermia rewarming. We found that except for 4 patients (cases 1, 2, 7, 9) whose GCS, APACHE II, SOFA, RASS scores had no significant relief compared with those before hypothermia therapy, the scores of the other 5 patients were significantly improved after hypothermia treatment. Therefore, high-quality individualized TTM therapy has great advantages in brain protection after CA.

**Table 3:** Disease severity score of each patient within 24 h before and after hypothermia treatment.

Variable	Patient								
	1	2	3	4	5	6	7	8	9
Pre-hypothermia									
GCS score	4	3	3	4	3	3	3	3	4
APACHE II score	32	29	29	20	29	30	32	22	40
SOFA score	14	12	10	14	12	15	10	11	13
RASS score	−3	−3	−3	−3	−3	−3	−3	−3	−3
Post-hypothermia									
GCS score	5	3	6	10	13	8	3	8	3
APACHE II score	34	32	14	16	7	14	35	19	40
SOFA score	15	10	7	10	1	5	14	9	13
RASS score	−3	−3	−3	−3	0	−2	−5	−1	−3

APACHE II = Acute physiology, Age and Chronic health evaluation, GCS = Glasgow coma scale, RASS = Richmond agitation-sedation scale, SOFA = sequential organ failure assessment.

The results of TCD, SSEP, EEG, imaging, and long-term follow-up are summarized in Table 4. In all patients, optic nerve sheath diameter (ONSD) was lower after hypothermia treatment, indicating that low temperature reduced intracranial pressure. Except for patient 2 who was forced to rewarm due to pulmonary infection, the increase of ONSD was not observed in other patients after rewarming. Based on TCD results, we found that the cerebral perfusion of patients with CA was either congested (cases 2, 3, 4, 6, 7, 9) or ischemic (cases 5, 8) prior to hypothermia treatment. In cases 6 and 9, CTA confirmed multiple intracranial vascular stenosis, increased cerebral blood flow velocity, and increased pulsatility index, which may be caused by vascular compliance. All the other patients benefited from hypothermia therapy and rewarmed successfully, except for cases 2 and 7 who had cerebral congestion again after rewarming and needed to be treated with hypothermia again. By EEG results, we found that patients with poor Synek grading before hypothermia therapy (cases 1, 2, 7) or patients with no improvement in Synek grading after hypothermia treatment (case 9) had a poor prognosis. However, the Synek grading of patients with good prognosis was significantly improved after individualized hypothermia treatment (cases 3, 4, 5, 6, 8), indicating that hypothermia treatment can ameliorate the symptoms of diffuse cerebral edema to a certain extent, thereby improving the brain function of patients. Previous studies have found that bilateral N20 disappearance often indicates poor prognosis in patients. In our study, N20 disappeared in 3 patients with poor long-term follow-up outcomes (cases 1, 2, 7), which is also consistent with previous findings. In addition, by comparing the brain imaging changes before and after hypothermia treatment, it was found that the gray matter ratio in the brain of patients with good prognosis was significantly improved compared with that before hypothermia treatment. Finally, in the long-term follow-up results 6 months after discharge, we found 3 patients (cases 2, 7, 9) died and 1 patient (case 1) had a long-term vegetative state. Among the patients with EEG reactivity, except for case 9, who died of bloodstream infection due to multiple underlying diseases, which was a non-neuro-related death, the other patients (cases 3, 4, 5, 6, 8) had a good prognosis and were able to work or study normally. It can be concluded that high-quality individualized TTM under the guidance of multi-mode brain monitoring plays a very important role in brain protection of patients with CA.

**Table 4:** MAP, TCD, EEG, SSEP, brain gray matter ratio, and long-term follow-up results of each patient before, during, and after high-quality individualized targeted temperature management.

	Patient								
	1	2	3	4	5	6	7	8	9
Pre-hypothermia									
MAP	73	86	103	79	82	86	89	83	80
ONSD	0.63	0.66	0.56	0.58	0.54	0.53	0.68	0.62	0.54
Vs	97	207	201	170	97	196	161	69	143
Vd	40	111	119	71	34	59	88	13	40
Vm	59	143	146	104	55	105	112	32	74
PI	0.96	0.66	0.61	0.96	1.14	1.31	0.66	1.76	1.39
CO	65.2	71.5	71.7	62.6	65	70.9	68.1	62	66.7
During hypothermia									
MAP	92	99	95	90	63	111	87	100	89
ONSD	0.62	0.52	0.54	0.51	0.51	0.55	0.58	0.58	0.53
Vs	118	113	86	77	116	124	143	78	178
Vd	59	54	34	42	40	33	67	32	50
Vm	79	74	51	54	65	63	92	47	93
PI	0.75	0.79	1.02	0.66	1.06	1.44	0.82	0.98	1.38
CO	63.9	63.7	62.9	59.9	66.2	61.8	65.4	64.3	60.3
Post-hypothermia									
MAP	96	94	77	86	73	115	108	97	106
ONSD	0.52	0.58	0.43	0.48	0.46	0.51	0.50	0.49	0.50
Vs	103	239	134	142	156	158	168	155	145
Vd	60	117	62	43	65	42	94	60	40
Vm	74	158	86	76	95	81	119	92	75
PI	0.75	0.78	0.84	0.82	0.96	1.44	0.94	1.03	1.40
CO	67.3	71.7	64.6	58.1	64.8	63.1	66.9	65.9	57.8
EEG Synek grading (pre/post-hypothermia)	IV-d/IV-d	IV-d/IV-d	II-a/I	II-a/I	III-b/I	II-a/I	IV-d/IV-d	II-a/I	III-d/III-d
EEG reactivity	-	-	+	+	+	+	-	+	+
Brain gray matter ratio	1.05/1/11	0.98/1.01	1.17/1.34	1.18/1.24	1.14/1.25	1.14/1.2	1.04/1.07	None/1.22	0.93/0/97
N20 amplitude (pre/post-hypothermia)	0/0	0/0	0.76/1.9	1.6/4.5	1.9/4.8	0.89/3.7	0/0	3.8/5.6	0.47/0.62
Long-term follow-up outcome									
mRS after 6 mo	5	6	1	1	0	2	6	3	6
GOS after 6 mo	2	1	5	5	5	5	1	4	1

CO = cerebral oxygenation, EEG = electroencephalogram, GOS = Glasgow outcome scale, MAP = mean arterial pressure, mRS = modified Rankin Scale, ONSD = optic nerve sheath diameter, PI = pulsatility index, Vd = end diastolic velocity, Vm = mean flow velocity, Vs = peak systolic velocity.

## 4. Discussion

Given the unpredictable nature of CA, which can occur at any time and place, the mortality rate is extremely high, even though patients who are successfully resuscitated from CA often have brain damage or systemic ischemia-reperfusion injury, post-traumatic stress symptoms, collectively known as post-CA syndrome.^[12]^ The first publication of articles on randomized controlled trials of hypothermia in 2002 led to the development of a series of guidance-based therapeutic cooling steps.^[13]^ TTM has also emerged as a treatment option after stroke, brain trauma, and CA.^[14]^ It is well known that patients who are resuscitated after CA often have a certain risk of neurological damage, such as the overproduction of free radicals and reactive oxygen species, apoptosis, destruction of the blood-brain barrier, brain edema, and among others after ischemia reperfusion. Hypothermia can reduce nerve oxygen consumption, improve glucose metabolism, and retain high phosphoric acid substrate to maintain a stable acid-base balance, thus playing a protective role in brain.^[15,16]^ Although current guidelines on TTM recommend a temperature setting of 32°C to 34°C, there is a large individual variation in efficacy and the long-term prognosis remains unclear. In a randomized controlled study, Nielsen et al^[17]^ concluded that in survivors of OHCA, a low temperature with a target temperature of 33°C did not provide any greater benefit than a target temperature of 36°C. A systematic review of 32 studies published from 2001 to 2021 concluded that the target of limiting temperature to 32°C to 34°C did not lead to improved 3 to 6 months survival or favorable functional outcomes after resuscitation compared to fever prevention.^[18]^ Another systematic review identified that different temperature targets, including hypothermia of 31°C to 32°C, 33°C to 34°C, and 35°C to 36°C, did not improve survival with good functional outcome as compared to normal temperatures of 37°C to 37.8°C after CA.^[10]^ Furthermore, there is also no uniform standard for the optimal duration of hypothermia treatment. The International Resuscitation Liaison Committee strongly recommends hypothermia for 12 to 24 hours in comatose patients with OHCA combined with ventricular fibrillation or non-perfusion ventricular tachycardia.^[19]^ In comatose survivors of OHCA, TTM at 33°C for 48 hours did not significantly improve long-term neurological outcomes compared to 24 hours of TTM at 33°C.^[20]^ As a result, there are still great inconsistencies and inaccuracies in the specific objectives and duration of temperature control for hypothermia therapy, and robust guidelines to help clinicians are sparse. According to the dynamic monitoring of the change of mean arterial pressure, clinically relevant score, SSEP, EEG, TCD results, we adjusted the target body temperature and developed an individualized cooling treatment plan.

In our study, based on the dynamic change of ONSD during hypothermia, we found that intracranial pressure was reduced during hypothermia regardless of long-term prognosis. Moreover, under high-quality individualized TTM treatment, patients not only relieve cerebral congestion or cerebral ischemia but also improve cerebral oxygen. In addition, N20 amplitude and Synek grade were also significantly improved in patients with good prognosis, and it was further found in brain imaging results that the gray matter ratio was higher than that before hypothermia treatment. It can be seen that the cerebral blood supply of patients was restored through high-quality individualized TTM treatment, and the expression of brain injury markers was improved at the same time. Through the results of rewarming and long-term follow-up, we found that in some patients with no EEG reactivity, even though the intracranial pressure and cerebral blood oxygen were temporarily improved during the hypothermia treatment, the TCD results of the patients showed that the brain was in ischemia again after rewarming, and the N20 amplitude was not relieved. This may be due to the lack of cerebrovascular autonomic regulation ability, so that patients eventually inevitably appear vegetative state or death. Therefore, we concluded that some patients can improve the cerebral blood supply state, restore the autonomic regulation ability of cerebrovascular, and improve the prognosis of patients through high-quality individualized TTM treatment, while others cannot benefit from hypothermia treatment and inevitably end up dying.

An inherent limitation of our study is the lack of controls due to the unpredictability of CA patients. Neurological outcomes after resuscitation from CA is a complex task for which ICU physician and neurologists need constant training. To minimize prognostic uncertainty, a multimodal brain monitoring approach is recommended for high-quality TTM in more patients. The small case presented in our study are intended to provide a novel literature basis for future experts to develop new guidelines to improve current clinical practice of TTM.

## Author contributions

**Data curation:** Weiping Xia, Meiling Ai, Xinhua Ma, Chenhuan Hu, Qianyi Peng, Chunguang Zhao, Qi Liu, Shixiong He.

**Formal analysis:** Weiping Xia, Xinhua Ma, Chenhuan Hu, Qianyi Peng, Chunguang Zhao, Qi Liu.

**Writing – original draft:** Weiping Xia, Meiling Ai.

**Investigation:** Xinhua Ma.

**Conceptualization:** Li Huang, Lina Zhang.

**Funding acquisition:** Li Huang.

**Writing – review & editing:** Li Huang.

## References

[1] Xu F Zhang Y Chen Y. Cardiopulmonary resuscitation training in China: current situation and future development. JAMA Cardiol. 2017;2:469–70. 28297007 10.1001/jamacardio.2017.0035

[2] Hoiland RL Robba C Menon DK Citerio G Sandroni C Sekhon MS. Clinical targeting of the cerebral oxygen cascade to improve brain oxygenation in patients with hypoxic-ischaemic brain injury after cardiac arrest. Intensive Care Med. 2023;49:1062–78. 37507572 10.1007/s00134-023-07165-x PMC10499700

[3] Henson T Rawanduzy C Salazar M. Outcome and prognostication after cardiac arrest. Ann N Y Acad Sci. 2022;1508:23–34. 34580886 10.1111/nyas.14699

[4] Girotra S Chan PS Bradley SM. Post-resuscitation care following out-of-hospital and in-hospital cardiac arrest. Heart. 2015;101:1943–9. 26385451 10.1136/heartjnl-2015-307450 PMC4780335

[5] Blanc A Colin G Cariou A. Targeted temperature management after in-hospital cardiac arrest: an ancillary analysis of targeted temperature management for cardiac arrest with nonshockable rhythm trial data. Chest. 2022;162:356–66. 35318006 10.1016/j.chest.2022.02.056

[6] Lüsebrink E Binzenhöfer L Kellnar A. Targeted temperature management in postresuscitation care after incorporating results of the TTM2 trial. J Am Heart Assoc. 2022;11:e026539. 36285786 10.1161/JAHA.122.026539 PMC9673653

[7] Callaway CW Donnino MW Fink EL. Part 8: post-cardiac arrest care: 2015 American Heart Association guidelines update for cardiopulmonary resuscitation and emergency cardiovascular care. Circulation. 2015;132:S465–482. 26472996 10.1161/CIR.0000000000000262 PMC4959439

[8] Aneman A Frost S Parr M Skrifvars MB. Target temperature management following cardiac arrest: a systematic review and Bayesian meta-analysis. Crit Care. 2022;26:58. 35279209 10.1186/s13054-022-03935-z PMC8917746

[9] Barker M Sekhon M Krychtiuk KA. Targeted temperature management after out-of-hospital cardiac arrest: integrating evidence into real world practice. Can J Cardiol. 2023;39:385–93. 36610519 10.1016/j.cjca.2022.12.026

[10] Fernando SM Di Santo P Sadeghirad B. Targeted temperature management following out-of-hospital cardiac arrest: a systematic review and network meta-analysis of temperature targets. Intensive Care Med. 2021;47:1078–88. 34389870 10.1007/s00134-021-06505-z

[11] Kalra R Arora G Patel N. Targeted temperature management after cardiac arrest: systematic review and meta-analyses. Anesth Analg. 2018;126:867–75. 29239942 10.1213/ANE.0000000000002646 PMC5820193

[12] Cunningham CA Coppler PJ Skolnik AB. The immunology of the post-cardiac arrest syndrome. Resuscitation. 2022;179:116–23. 36028143 10.1016/j.resuscitation.2022.08.013

[13] Mild therapeutic hypothermia to improve the neurologic outcome after cardiac arrest. N Engl J Med. 2002;346:549–56. 11856793 10.1056/NEJMoa012689

[14] Huang J Wang P Wen H. The safety and efficacy of hypothermia combining mechanical thrombectomy or thrombolysis in the treatment of ischemic stroke: a systematic meta-analysis. Clinics (Sao Paulo). 2023;78:100218. 37269787 10.1016/j.clinsp.2023.100218 PMC10242631

[15] Guan L Lee H Geng X. Neuroprotective effects of pharmacological hypothermia on hyperglycolysis and gluconeogenesis in rats after ischemic stroke. Biomolecules. 2022;12:851. 35740974 10.3390/biom12060851 PMC9220898

[16] Belur AD Sedhai YR Truesdell AG. Targeted temperature management in cardiac arrest: an updated narrative review. Cardiol Ther. 2023;12:65–84. 36527676 10.1007/s40119-022-00292-4 PMC9986171

[17] Nielsen N Wetterslev J Cronberg T.; TTM Trial Investigators. Targeted temperature management at 33°C versus 36°C after cardiac arrest. N Engl J Med. 2013;369:2197–206. 24237006 10.1056/NEJMoa1310519

[18] Sandroni C Natalini D Nolan JP. Temperature control after cardiac arrest. Crit Care. 2022;26:361. 36434649 10.1186/s13054-022-04238-z PMC9700892

[19] Nielsen N Friberg H. Changes in practice of controlled hypothermia after cardiac arrest in the past 20 years: a critical care perspective. Am J Respir Crit Care Med. 2023;207:1558–64. 37104654 10.1164/rccm.202211-2142CP

[20] Kirkegaard H Søreide E de Haas I. Targeted temperature management for 48 vs 24 hours and neurologic outcome after out-of-hospital cardiac arrest: a randomized clinical trial. JAMA. 2017;318:341–50. 28742911 10.1001/jama.2017.8978 PMC5541324

